# Work fatigue among Lebanese physicians and students during the COVID-19 pandemic: validation of the 3D-Work Fatigue Inventory (3D-WFI) and correlates

**DOI:** 10.1186/s12889-022-12733-9

**Published:** 2022-02-12

**Authors:** Elsa Sfeir, Jean-Marc Rabil, Sahar Obeid, Souheil Hallit, Marie-Claude Fadous Khalife

**Affiliations:** 1 School of Medicine and Medical Sciences, Holy Spirit University of Kaslik, P.O. Box 446, Jounieh, Lebanon; 2 Social and Education Sciences Department, School of Arts and Sciences, Lebanese American University, Jbeil, Lebanon; 3 Research Department, Psychiatric Hospital of the Cross, Jal-Eddib, Lebanon; 4 Department of Pediatrics, Notre Dame des Secours University Hospital Center, Street 93, Byblos, Postal Code 3 Lebanon

**Keywords:** Work fatigue, Physicians, Residents, Interns, COVID-19 pandemic, Lebanon

## Abstract

**Background:**

Work fatigue is a work-related condition that affects physicians’ health, work attitude safety and performance. Work fatigue affects not only medical workers but can also leave a negative impact on patients. With the burden of the COVID-19 pandemic as well as the economic crisis Lebanese doctors have been facing in the last 2 years, the aim of our study was to validate the 3D-Work Fatigue Inventory (3D-WFI) among Lebanese physicians and assess the rate and correlates of work fatigue (physical, mental and emotional).

**Methods:**

A cross-sectional study was undertaken through an anonymous self-administered questionnaire between October 2020 and January 2021. The SPSS AMOS software v.24 was used to conduct confirmatory factor analysis (CFA). To validate the 3D-WFI, multiple indices of goodness-of-fit were described: the Relative Chi-square (χ2/df) (cut-off values:< 2–5), the Root Mean Square Error of Approximation (RMSEA) (close and acceptable fit are considered for values < 0.05 and < 0.11 respectively), the Tucker Lewis Index (TLI) and the Comparative Fit Index (CFI) (acceptable values are ≥0.90).

**Results:**

A total of 401 responses was collected; 66.1, 64.8 and 65.1% respondents had an intermediate to high level of emotional, mental and physical work fatigue respectively. The fit indices obtained in the CFA of the 3D-WFI items fitted well: CFI =0.98, TLI =0.98, RMSEA = 0.05; 95% CI 0.046–0.063; pclose = 0.20) and χ^2^(136) = 295.76. The correlation coefficients between the three factors (Factor 1 = Physical work fatigue, Factor 2 = Mental work fatigue, Factor 3 = Emotional work fatigue) were adequate as well: Factor 1-Factor 2 (*r* = 0.70), Factor 1-Factor 3 (*r* = 0.52) and Factor 2-Factor 3 (*r* = 0.65). In addition, feeling pressured by long working hours during the pandemic, having more hours of night duty per month, more stressful events in life, and higher depression were significantly associated with more physical and mental work fatigue. Higher depression and having more stressful events in life were significantly associated with more emotional work fatigue.

**Conclusion:**

Work fatigue in Lebanese physicians seems to be associated with higher level of everyday stress, high work load and depression. Hospitals and local health authorities can use these results for early interventions that aim to reduce work fatigue and ensure the wellbeing of Lebanese physicians.

## Background

Work fatigue is viewed as a personal and work-related condition that links employees’ health, work attitude, safety and performance to work condition [1]. Work fatigue is associated with extreme exhaustion and tiredness with diminished working capacity that is felt during and at the end of working days.

Work fatigue is divided into 3 main features: physical, mental and emotional work fatigue [1]. Physical work fatigue is defined as an extreme physical tiredness and reduced capacity to engage in physical activity, while emotional fatigue is an emotional tiredness and reduced ability to engage in emotional activities at the end or during working days. Finally, mental work fatigue relates to cognitive tiredness that prevents workers from engaging in cognitive activities during or at the end of working days. Chronic work fatigue can lead by itself to burnout with loss of personal achievements [2].

Work fatigue in medical professionals has received a great amount of attention in recent years after many studies showed that this population is particularly vulnerable for developing mental, emotional and physical exhaustion [3–5]. Studies among medical professionals reported moderate-to-high levels of emotional exhaustion and depersonalization, with low-to-moderate levels of personal achievements [6–8]. In addition, high work fatigue affects health and wellbeing of doctors [7].

The consequences of work fatigue are not limited to the affected medical workers themselves, but they can leave a negative impact on patients and their medical practice, and can consequently increase the number of medical errors. For instance, higher mental and physical exhaustion in residents and physicians was associated with higher rate of medical errors [9]. Consequently, work fatigue is considered as a public health problem that can leave a negative impact on individual physicians, patients and healthcare organizations and system [10].

Many factors have been previously described to be associated with higher work fatigue in medical professionals. For example, stressful events in life were described to be associated with higher levels of work fatigue and were a predisposing event to many mental morbidities [5, 11]. On another hand, long night shifts were shown to keep a negative impact on doctors’ mental health and emotional wellbeing [12]. Higher overall fatigue in residents was associated with an increase number of duties per month [13]. Similarly, burnout syndrome has been previously described to vary between medical specialties [13]. For instance, family medicine physicians were found to score higher rates of work fatigue and burnout compared to other specialties. Those rates could be attributed to higher workload with lowest cost by service [14]. In addition, medical professionals were shown to have different levels of emotional fatigue depending on work conditions and demands [15]. In other words, work conditions can directly affect work fatigue. In fact, it was previously described that high workload, more time pressure, long working hours [15], violence, terror and conflicts at work [16] were shown to be associated with higher level of exhaustion and work fatigue. A study conducted in Sweden showed that policy changes, budget cuts as well as reorganization, were factors predisposing to higher rate of work fatigue and exhaustion in medical professionals [17]. In addition, medical professionals with somatic or mental disease comorbidities were more vulnerable for developing exhaustion and are consequently at a higher risk for developing work fatigue syndrome [17]. In fact, medical professionals having depression or anxiety were shown to be more affected by work fatigue and exhaustion and were at a higher risk of recurrence of work fatigue syndrome [18, 19].

The novel coronavirus has been described as a source of fear and phobia in the workplace of medical professionals since December 2019. Consequently, it was considered as a novel stressor affecting work fatigue, mental health and wellbeing [20]. The fear of COVID-19 was positively associated with higher levels of work place panic and avoidance behaviors. Consequently, medical doctors working during this pandemic showed higher work place phobia, emotional exhaustion leading to worse performance at work [21, 22]. Additionally, the COVID-19 bio-disaster has proven to carry wider psychosocial repercussions on healthcare workers, with significantly high prevalence of depression, anxiety, PTSD, insomnia, distress and burnout as shown by the meta-analysis conducted by Batra et al. [23]. This increased incidence of psychosocial dysfunction seems to have carried over to students, as demonstrated by another large meta-analysis involving 90,879 college students, where depression, stress and anxiety have significantly increased when compared to their pre-COVID-19 incidence rates [24].

Lebanese physicians have been previously shown to score high rates of work fatigue and exhaustion in comparison to the general population [25, 26]. In addition, the past year was particularly challenging for Lebanese medical doctors and students, owing to a whirlwind of tumultuous events, most prominent of which being the COVID-19 pandemic. Doctors shouldered the risk of treating COVID-19 patients in often unsafe conditions and insufficient personal protective equipment material in Lebanon, especially in the early months of the pandemic, during which many lives from the medical corps were lost to this lethal virus [27]. The pandemic itself came on the backdrop of a crushing economic crisis with a plummeting of the local currency’s value and the highest inflation rate the country has known in more than 30 years [28]. Finally, an explosion of massive proportions, on the 4th of August 2020, rocked the capital city causing more than two hundred deaths and thousands of injuries, including medical doctors, residents and medical students working in nearby hospitals [29]. Evidently, these crises were an additional source of increased work-related stress and would suggest an increase in work fatigue rate in this very susceptible population.

Fatigue can have multiple aspects in the work place. Consequently, measuring work fatigue can be challenging and appears not to have a single tool to assess it [30]. The three dimension-work fatigue inventory (3D-WFI) has been used and validated to assess work fatigue. It provides a full and commensurate assessment of emotional, physical and mental work fatigue [30]. To our knowledge, in the Arabic speaking population, there has been no published study reporting the validation of the work fatigue inventory. The two validated Arabic assessment measurement are the Arabic Version of the Copenhagen Psychosocial Questionnaire II (A-COPSOQ II) [31] and the brief fatigue inventory [32]. However, those inventories are not multifactorial and do not appear to cover the three dimensions of work fatigue. Therefore, the validation of the 3D-WFI in Lebanon seems warranted.

To the best of our knowledge, no recent studies have been conducted to gauge the extent of the damage incurred on the Lebanese medical population after the events of the past year in terms of physical work and general mental health. In this context, our main aim was to validate the 3D-Work Fatigue Inventory among Lebanese physicians and assess the rate and correlates of work fatigue (physical, mental and emotional).

## Methods

### Study design

A cross-sectional study was undertaken through an anonymous self-administered questionnaire of 104 questions created using Kobo Toolbox and sent online on WhatsApp at different hospitals in the country (Notre Dame Des Secours Hospital, Eye and Ear Hospital, Psychiatric Hospital of the Cross, Beit Chabab Hospital, Saint George Hospital University Medical Centre, Hotel Dieu de France Hospital, and AUB Medical Center). Dissemination of the survey was done through the snowball sampling method. Each member of the research team contacted interns, residents and physicians he/she knows and asked him/her to fill the survey. Participants were then asked to send the link to other potential people who might be eligible for participation. This process was continued until the minimal sample size was reached. Four hundred and one answers were obtained between October 2020 and January 2021. Every Lebanese doctor, resident and intern working in Lebanon was eligible to participate (the sample includes both physicians and physicians-in-training; in this paper, we will refer to the collective group as “physicians” for ease of interpretation). Excluded were Lebanese physicians or physicians-in-training practicing outside Lebanon, and doctors working in a non-clinical and purely academic capacity.

### Participants

Our sample encompassed Lebanese MED-3 and MED-4 students or their equivalents in 6th and 7th medical year from universities following the French system, as well as residents and attending physicians from 34 medical specialties practicing in Lebanon during the last year. Residents and attending physicians were divided into 3 groups according to their specialty: medical specialties (Pulmonology and Critical Care, Cardiology, Infectious Disease, Neurology, Internal Medicine, General Pediatrics, Hematology and Oncology, Emergency Medicine, Nephrology, Gastro-enterology and General Medicine), surgical specialties (Obstetrics and Gynecology, Orthopedic Surgery, Pediatric Surgery, General Surgery, Urology, Neurosurgery, Cardiothoracic Surgery and Vascular Surgery) and other specialties (Psychiatry, Anesthesiology, Ophthalmology, ENT, Radiology, Dermatology, Endocrinology, Plastic Surgery, Family Medicine, Pathology and Histology, Laboratory Medicine, Interventional Radiology, Allergology and Immunology and Occupational Medicine). This was established on the basis of these specialties providing (1) a good degree of work-life balance, (2) a less critically ill patient pool in general, (3) a good degree of work site or clinical predictability, (4) defined and focused limits of the necessary knowledge base, (5) limited contact with patients, (6) good degree of control over work schedules and finally (7) specialties that are traditionally very well remunerated in Lebanon. Most of the specialties included in the third group satisfy more than three of these conditions.

### Minimal sample size calculation

Referring to the G*power 3.1.9.7 software (multiple regression: R^2^ deviation from zero) [33, 34], a minimal number of 389 physicians was required to secure significance when considering the following statistical parameters: type I error α = 5%, power 1-β = 80%, a small effect size f^2^ = 5%, and a total number of 15 variables to be integrated in the multivariable analysis.

Regarding the minimal sample size to perform a confirmatory factor analysis, a minimal sample of 360 participants was deemed necessary to validate the 3D-WFI scale, based on 20 participants per 1 scale item [35].

### Measures

The questions contained in this online form pertained to general socio-demographic factors (age, gender, monthly income, and living situation), a section to quantify the workload, and a section about associated factors such as depression (measured by the Patient Health Questionnaire (PHQ-9) scale). This scale is composed of 9 items (e.g. “Over the last 2 weeks, how often have you been bothered by any of the following problems? Feeling tired or having little energy”), scored from 0 = not at all to 3 = nearly every day [36]. This scale is validated in Lebanon [37]. Higher scores indicate more depressive symptoms (in this study, Cronbach’s alpha = 0.872).

Work fatigue was assessed using the 3D-WFI, divided into three dimensions: physical (e.g. “During the PAST 12 MONTHS, how often did you feel physically exhausted at the end of the workday?”), mental (e.g. “During the PAST 12 MONTHS, how often did you feel mentally exhausted at the end of the workday?”) and emotional fatigue (e.g. “During the PAST 12 MONTHS, how often did you feel emotionally exhausted at the end of the workday?”) [1]. Each domain is composed of 6 questions, with higher scores reflecting higher work fatigue in all domains (in this study, Cronbach’s alpha were 0.957, 0.954 and 0.966 for the physical, mental, and emotional subscales respectively).

A score reflecting stressful events in life during the last year based on the answers (yes/no) to 5 questions about death in the family, divorce, financial loss, romantic failure and proximity to the port explosion (within a 2 Km radius) (in this study, Cronbach’s alpha = 0.712).

The fear from the coronavirus was calculated using the Fear of COVID-19 scale [38]. The latter is validated in Arabic [39] and is composed of 7 items (e.g. “It makes me uncomfortable to think about Corona”), with higher scores reflecting more fear from the coronavirus (in this study, Cronbach’s alpha = 0.932).

### Statistical analysis

The SPSS AMOS software v.24 was used to conduct confirmatory factor analysis for the 3D-WFI. Multiple indices of goodness-of-fit were described: the Relative Chi-square (χ2/df) (cut-off values:< 2–5), the Root Mean Square Error of Approximation (RMSEA) (close and acceptable fit are considered for values < 0.05 and < 0.11 respectively), the Tucker Lewis Index (TLI) and the Comparative Fit Index (CFI) (acceptable values are ≥0.90) [40].

The SPSS software v.25 was used to conduct the statistical analysis. The three 3D-WFI scores were normally distributed as verified by their skewness and kurtosis values, which varied between − 2 and + 2 [41]. The Student t test was used to compare two means, whereas the ANOVA test was used to compare three or more means. Pearson correlation test was used to correlate two continuous variables. Effect sizes were calculated for all bivariate analyses; in psychological research, values of 0.1 were deemed to have small effect size, whereas values of 0.2 and 0.3 were classified as having medium and large effect sizes respectively [42, 43]. Linear regressions were conducted taking each work fatigue subscale score as the dependent variable. Independent variables that showed correlation coefficients or effect sizes ≥ │0.24│ were entered in the linear regressions to have more parsimonious models [44]. Significance was set at *p* < 0.05.

## Results

The sociodemographic and other characteristics of the participants are summarized in Table 1. The mean age was 34.50 ± 13.48 years, with 57.9% males. Moreover, 107 (26.7%) had high physical activity, whereas 122 (30.4%) and 103 (25.7%) had high mental and emotional work fatigue respectively.

**Table 1:** Sociodemographic and other characteristics of the participants (*N* = 401)

Variable	N (%)
**Gender**	
Male	232 (57.9%)
Female	169 (42.1%)
**Marital status**	
Single/widowed/divorced	272 (67.8%)
Married	129 (32.2%)
**Living situation**	
With the family	312 (77.8%)
With a flat mate	27 (6.7%)
Alone	62 (15.5%)
**Medical rank**	
Attending physician	149 (37.2%)
Resident	149 (37.2%)
Intern	103 (25.7%)
**Specialty**	
Medical	101 (25.2%)
Surgical	97 (24.2%)
Other	203 (50.6%)
**Monthly income (in LBP)**	
< 3 million	212 (52.9%)
Between 3 and 10 million	78 (19.5%)
> 10 million	111 (27.7%)
**Most cause of work difficulty**	
Economic instability	150 (37.4%)
Political instability	27 (6.7%)
COVID-19	158 (39.4%)
Long working hours	36 (9.0%)
Increased administrative tasks	25 (6.2%)
Patient attitude towards personnel	5 (1.2%)
**WFI physical score categories**	
Low (≤17)	140 (34.9%)
Moderate (18–24)	154 (38.4%)
High (≥25)	107 (26.7%)
**WFI mental score categories**	
Low (≤16)	141 (35.2%)
Moderate (17–23)	138 (34.4%)
High (≥24)	122 (30.4%)
**WFI emotional score categories**	
Low (≤15)	136 (33.9%)
Moderate (16–24)	162 (40.4%)
High (≥25)	103 (25.7%)
	**Mean ± SD**
Age (in years)	34.50 ± 13.48
Number of children	0.56 ± 1.05
Working hours per day	8.99 ± 1.88
Working hours per week	60.56 ± 17.24
Number of hours of night duty per month	64.91 ± 74.14

*LBP* Lebanese pounds, *WFI* Work fatigue inventory

### Confirmatory factor analysis of the work fatigue inventory

The fit indices of the CFA model from the sample in this study fitted well: CFI =0.98, TLI =0.98, RMSEA = 0.05; 95% CI 0.046–0.063; pclose = 0.20) and χ^2^(136) = 295.76. The correlation coefficients between the three factors (Factor 1 = Physical work fatigue, Factor 2 = Mental work fatigue, Factor 3 = Emotional work fatigue) were adequate as well: Factor 1-Factor 2 (*r* = 0.701), Factor 1-Factor 3 (*r* = 0.519) and Factor 2-Factor 3 (*r* = 0.651).

**Fig. 1:**
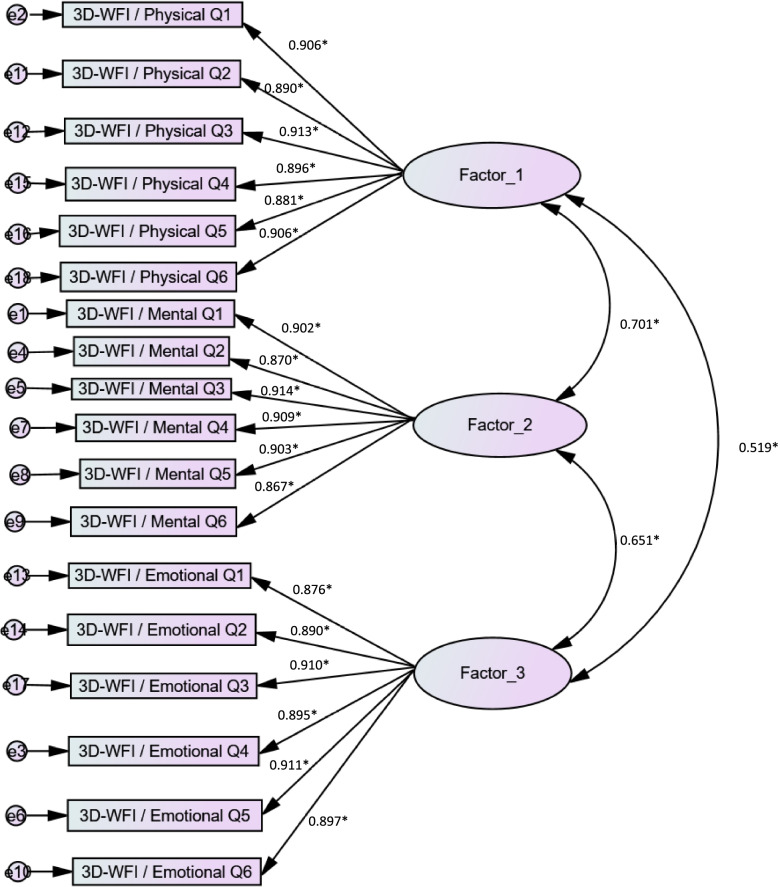
Standardized factor loadings of three-factor model of the Arabic version of the 3D-Work Fatigue Inventory scale. *p < 0.001

The standardized factor loadings of the three-factor model of the Arabic version of the 3D-WFI scale are presented in Fig. 1.

### Bivariate analysis

Higher mean physical and mental work fatigue was significantly found in single participants compared to married ones, in interns compared to attending physicians and residents, in those who have a monthly income more than 10 million LBP, in those who feel pressured by long working hours. Feeling pressured by the COVID-19 pandemic was significantly associated with more mental work fatigue. Finally, higher mean emotional work fatigue was significantly found in interns compared to attending physicians and residents, and in those who feel pressured by long working hours (Table 2).

**Table 2:** Bivariate analysis of categorical variables associated with physical, mental, and emotional work fatigue scores

Variable	Physical work fatigue	***p***	Effect size	Mental work fatigue	***p***	Effect size	Emotional work fatigue	***p***	Effect size
**Gender**		0.883	0.014		0.958	0.005		0.601	0.052
Male	20.17 ± 5.95			19.39 ± 6.00			19.28 ± 6.74		
Female	20.08 ± 6.07			19.36 ± 5.76			18.93 ± 6.67		
**Marital status**		**< 0.001**	0.463		**< 0.001**	0.454		0.09	0.182
Single/ widowed /divorced	21.01 ± 5.84			20.22 ± 5.77			19.53 ± 6.68		
Married	18.29 ± 5.90			17.60 ± 5.77			18.31 ± 6.70		
**Living situation**		0.175	0.093		0.383	0.069		0.833	0.030
With the family	20.25 ± 5.79			19.41 ± 5.85			19.22 ± 6.71		
With a flat mate	21.41 ± 6.07			20.56 ± 5.75			19.26 ± 6.10		
Alone	19.02 ± 6.86			18.69 ± 6.18			18.66 ± 6.99		
**Medical rank**		**< 0.001**	0.336		**< 0.001**	0.346		**< 0.001**	0.225
Attending physician	18.21 ± 6.15			17.60 ± 6.04			19.27 ± 6.84		
Resident	20.01 ± 5.62			19.01 ± 5.37			17.52 ± 6.31		
Intern	23.10 ± 5.08			22.49 ± 5.19			21.28 ± 6.49		
**Specialty**		0.665	0.045		0.145	0.098		0.448	0.063
Medical	19.96 ± 6.12			19.15 ± 5.90			18.55 ± 7.02		
Surgical	19.77 ± 5.97			18.52 ± 6.07			18.90 ± 6.71		
Other	20.39 ± 5.96			19.91 ± 5.77			19.54 ± 6.54		
**Monthly income (in LBP)**		**< 0.001**	0.252		**< 0.001**	0.244		0.545	0.055
< 3 million	21.52 ± 5.53			20.69 ± 5.56			19.30 ± 6.65		
Between 3 and 10 million	18.65 ± 6.05			18.23 ± 6.02			18.38 ± 6.62		
> 10 million	18.53 ± 6.20			17.68 ± 5.86			19.35 ± 6.89		
**Pressure from economic instability**		0.196	0.133		0.577	0.057		0.483	0.073
No	20.43 ± 5.91			19.51 ± 5.79			19.32 ± 6.36		
Yes	19.63 ± 6.11			19.17 ± 6.07			18.82 ± 7.26		
**Pressure from political instability**		0.699	0.072		0.793	0.055		0.078	0.361
No	20.17 ± 5.92			19.36 ± 5.94			18.98 ± 6.71		
Yes	19.70 ± 7.05			19.67 ± 5.23			21.33 ± 6.29		
**Pressure from the COVID pandemic**		0.075	0.184		**0.010**	0.266		0.063	0.189
No	20.56 ± 6.15			19.99 ± 5.98			19.63 ± 6.97		
Yes	19.47 ± 5.70			18.44 ± 5.64			18.38 ± 6.22		
**Pressure from long working hours**		**< 0.001**	0.888		**< 0.001**	1.006		**0.01**	0.442
No	19.73 ± 6.02			18.93 ± 5.87			18.88 ± 6.74		
Yes	24.22 ± 3.86			23.92 ± 3.83			21.67 ± 5.86		
**Pressure from increased administrative tasks**		0.220	0.252		0.430	0.163		0.430	0.167
No	20.04 ± 5.98			19.32 ± 5.90			19.07 ± 6.73		
Yes	21.56 ± 6.08			20.28 ± 5.89			20.16 ± 6.33		

Numbers in bold indicate significant *p*-values

Higher physical work fatigue was significantly associated with younger age, a lower number of children, more working hours per week, more hours of night duty per month, a higher number of comorbidities, more stressful events in life and depression.

Higher mental work fatigue was significantly associated with younger age, a lower number of children, mroe working hours per day or per week, more hours of night duty per month, a higher number of comorbidities, more stressful events in life and depression.

Higher emotional work fatigue was significantly associated with a higher number of comorbidities, more stressful events in life, more depression and more fear of COVID-19 (Table 3).

**Table 3:** Bivariate analysis of continuous variables associated with physical, mental, and emotional work fatigue scores

Variable	Physical work fatigue	Mental work fatigue	Emotional work fatigue
Age	−0.227^***^	−0.228^***^	0.004
Number of children	−0.114	− 0.129^*^	0.017
Working hours per day	0.065	0.102^*^	0.005
Working hours per week	0.158^**^	0.166^**^	0.050
Night duty hours per month	0.316^***^	0.204^***^	0.052
Number of comorbidities	0.124^*^	0.108^*^	0.222^***^
Stressful events score	0.241^***^	0.272^***^	0.370^***^
Depression (PHQ-9 score)	0.445^***^	0.464^***^	0.491^***^
Fear of COVID-19 score	0.013	0.051	0.133^**^

^*^ *p* < 0.05; ^**^ *p* < 0.01; ^***^ *p* < 0.001; numbers refer to Pearson correlation coefficients

### Multivariable analysis

The results of a first linear regression, taking the physical work fatigue score as the dependent variable, showed that feeling pressured by long working hours during the pandemic (Beta = 2.58), having more hours of night duty per month (Beta = 0.02), more stressful events in life (Beta = 0.93), and higher depression (Beta = 0.38) were significantly associated with more physical work fatigue (Table 4, Model 1).

**Table 4:** Multivariable analyses

Variable	Unstandardized Beta	Standardized Beta	***p***	95% CI
**Model 1: Linear regression taking the physical work fatigue as the dependent variable.**				
Marital status (married vs single^a^)	0.15	0.01	0.852	−1.40-1.70
Resident vs attending physician^a^	0.71	0.06	0.557	−1.67-3.10
Intern vs attending physician^a^	2.21	0.16	0.097	−0.40-4.81
Monthly income (intermediate vs low^a^)	−0.38	− 0.03	0.673	−2.15-1.39
Monthly income (high vs low^a^)	1.00	0.08	0.416	−1.42-3.42
Pressure from long working hours during the pandemic (yes vs no^a^)	2.58	0.12	**0.007**	0.73–4.44
Pressure from increased administrative tasks during the pandemic (yes vs no^a^)	−0.24	−0.01	0.829	−2.44-1.95
Number of night duty hours per month	0.02	0.24	**< 0.001**	0.01–0.03
Stressful events in one’s life	0.93	0.14	**0.002**	0.36–1.51
Depression	0.38	0.34	**< 0.001**	0.28–0.48
Nagelkerke *R*^2^ = 32.6%				
**Model 2: Linear regression taking the mental work fatigue as the dependent variable.**				
Marital status (married vs single^a^)	−0.02	−0.001	0.983	−1.56-1.52
Resident vs attending physician^a^	1.29	0.11	0.266	−0.99-3.56
Intern vs attending physician^a^	2.40	0.18	0.064	−0.14-4.93
Monthly income (intermediate vs low^a^)	−0.02	−0.002	0.980	−1.76-1.72
Monthly income (high vs low^a^)	0.35	0.03	0.773	−2.06-2.76
Pressure from the coronavirus during the pandemic (yes vs no^a^)	−0.16	−0.01	0.785	−1.27-0.96
Pressure from the long working hours during the pandemic (yes vs no^a^)	3.71	0.18	**< 0.001**	1.85–5.56
Stressful events in one’s life	1.27	0.20	**< 0.001**	0.70–1.83
Depression	0.41	0.37	**< 0.001**	0.31–0.51
Nagelkerke *R*^2^ = 31.8%				
**Model 3: Linear regression taking the emotional work fatigue as the dependent variable.**				
Pressure from the political instability in the country (yes vs no^a^)	1.44	0.05	0.228	−0.91-3.79
Pressure from the long working hours during the pandemic (yes vs no^a^)	1.97	0.08	0.053	−0.03-3.98
Stressful events in one’s life	2.06	0.28	**< 0.001**	1.43–2.68
Depression	0.54	0.43	**< 0.001**	0.43–0.65
Nagelkerke *R*^2^ = 32.6%				

^a^Reference group

The results of a second linear regression, taking the mental work fatigue score as the dependent variable, showed that feeling pressured by long working hours during the pandemic (Beta = 3.71), more stressful events in life (Beta = 1.27), and higher depression (Beta = 0.41) were significantly associated with more mental work fatigue (Table 4, Model 2).

The results of a third linear regression, taking the emotional work fatigue score as the dependent variable, showed that higher depression (Beta = 0.54) and having more stressful events in life (Beta = 2.06) were significantly associated with more emotional work fatigue (Table 4, Model 3).

## Discussion

The results of our study were alarming, showing high rates (intermediate-to-high) of emotional (66.1%), physical (65.1%) and mental (64.8%) work fatigue in Lebanese doctors. These results were higher than previously cited work fatigue rates among Lebanese physicians. It has been previously shown that 37.2% of Lebanese physicians had emotional work fatigue in 2018 [45]. Our results were also higher than the ones found in international data, showing a rate of 45.8% in US physicians and 43.5% of European physicians [46, 47]. A previous study conducted on Lebanese training doctors (residents) showed high level of exhaustion reaching a rate of 80% [16], and 37.5% in Lebanese Doctors in 2019 [45]. Similarly, when comparing those rates with the rate of work fatigue in Arab countries, we find that the rates found in our study were much higher. In 2018, doctors working in Saudi Arabia showed higher rates of emotional work fatigue in comparison with Lebanese doctors [48]. This difference was previously attributed to higher rates of tolerance in Lebanese doctors. The difference in these results (higher levels of work fatigue found in Lebanese doctors in comparison with other Arab doctors) could be attributed to higher levels of daily stressors faced by Lebanese doctors during the last year such as the economic crisis and Beirut blast. In addition, COVID-19 pandemic exposing Lebanese doctors to higher working hours, more stressful work conditions and a higher number of night shifts per month can be another reason behind those high level of work fatigue [49].

The confirmatory analysis results showed that the Arabic version of the 3D-WFI consisted of 3 dimensional structures including mental, physical and emotional fatigue, similar to the factor structure obtained in the original paper [1]. Furthermore, the correlation coefficients between the three factors were adequate as well. Confirmatory factors analysis showed that the Lebanese version of the 3D-WFI showed a good internal consistency as well, similar to the original version. The initial psychometric properties of the scale indicate that the Arabic version of the 3D-WFI is a reliable scale to measure work fatigue, with further studies warranting more psychometric properties (test-retest, convergent validity, etc.).

In our study, higher stressful events in life were associated with higher emotional, mental and physical work fatigue in Lebanese physicians [50]. Chronic and acute interpersonal stressors were associated with higher work fatigue [51]. In addition, being exposed to a major stressful event has been previously associated with higher work fatigue in doctors [45]. Lebanese physicians were described to be prone to high levels of daily stress giving a high threshold of tolerance and coping strategies [45]. Economic instability, terror at work, as well as economic instability [52] were described to be associated with higher rates of work fatigue in medical professionals [16]. Furthermore, the COVID-19 pandemic was a major contributor for increased stressful events in the Lebanese general population [53]. The fear of contracting the disease given the direct role doctors play in treating and diagnosing the virus, the higher rate of workload, as well as the lack of mental and social support physician were facing, were described to be associated with higher rate of emotional, physical and mental work fatigue among doctors [21]. In other words, COVID-19 phobia was cited to be a major contributor for higher levels of depression, anxiety and work fatigue in medical workers [21, 54]. Many factors were cited to contribute to excessive fatigue and exhaustion such as work overload, frequent overtime, work pressure and stress about work has to be done [55, 56]. Consequently, strategies to reduce the workload and fatigue were mandatory in hospital physicians during COVID-19 pandemic [56].

In the same perspective, higher night duty hours per month during COVID-19 pandemic were shown to be associated with higher physical and mental work fatigue in our study. Those results were in agreement with previous findings showing that longer night shifts and a higher workload were predisposing factors for mroe work fatigue [55]. In fact, the past 2 years have been challenging for Lebanese doctors given the burden of the COVID-19 pandemic that led to extended shifts duration leading to sleep deprivation in working physicians and a reduction in resting time, which can consequently affect the physician’s performance following physical and mental exhaustion [57]. This was seen in Lebanese physicians working more than 60 working hours per week [45]. In addition, decreasing working hours per week was shown to lower work fatigue and exhaustion. Indeed, increasing doctors sleeping hours and vacation time has been described to be a protective factor against work fatigue [58–60].

We showed in our study that higher rates of depression were associated with higher rates of physical, mental and emotional work fatigue, in agreement with a Chinese study [7]. In addition, another study conducted in Swedish primary care showed that depression was a predisposing factor for higher level of work fatigue and exhaustion [17]. As depression reduces a person’s ability to cope and deal with stress, it can enhance this imbalance, leading to higher rates of exhaustion and work fatigue [17]. Consequently, identifying symptoms of depression seems mandatory in order to prevent and reduce the burden of work fatigue and exhaustion syndrome in healthcare workers [17].

### Clinical implications

Work fatigue is a serious problem affecting doctors and physician in their everyday life. The impact it can leave on physicians’ mental health, work attitude and consequently medical errors, makes of it a serious public health issue. With the alarming rates of work fatigue among Lebanese physicians, further recognition of work fatigue risk factors is an important first step in order to prevent and treat work fatigue in doctors. Having a validated tool to assess work fatigue among physician is an important step since assessment of work fatigue is the first step towards prevention strategies.

This study provides much-needed appraisal of the extent of the mental health crisis that the Lebanese medical population has been living through during the last 2 years. The inclusion of physicians from all specialties, as well as future physicians in residents and medical students, showed a medical workforce that has severely suffered from work fatigue during the COVID-19 pandemic, throughout its ranks and fields. Considering the proven deleterious psychosocial impact of work fatigue, this article should sound alarm bells for the Lebanese government to urgently take action by implementing prospective mental health and well-being interventions to all medical personnel, such as education on coping techniques, online wellness activities and fostering post-traumatic growth. Additionally, this article endorses duty hour reforms and more stringent control over hospital policies for working schedules. This is especially needed for medical students and residents who suffer most from long working hours, overnight shifts and a lax application of shift length regulations.

### Limitations

This is a cross sectional study; consequently, causality effect between work fatigue and associated factors cannot be established. In addition, comparing work fatigue rate to previous data can be slightly imprecise given that different assessment tools were used in different studies. The snowball technique used during the data collection predisposes us to a selection bias. Multiple responses from the same participant could not be prevented as this issue cannot be controlled through online forms. Some specialties in Lebanese doctors were under or over presented. The questionnaire assessed common work environment consequently the assessment will not be precise as if targeting each specialty alone. We did not use a validated scale to measure stress. Information bias is possible since doctors might report their symptoms in an erroneous way. Other factors associated with work fatigue were not assessed in this study, predisposing us to a possible confounding bias. The sample is too small to allow for a measurement invariance between genders and between professional titles. The lack of Rasch analysis to confirm the ordinal nature of the items was not done as well. Although the fit indices of the CFA were good, some authors argued about the reliability of these thresholds to determine a good or bad fit of a scale; large samples can bias the Chi-square [61], RMSEA [62, 63], CFI and TLI values [64]. Consequently, those findings should be interpreted with caution.

## Conclusion

Having a validated tool to assess work fatigue such as the 3D-WFI tool in all its dimensions is useful for epidemiological studies in the country. Lebanese physicians scored alarming rates of work fatigue in its three domains. Stressors in life, depression as well as difficult work conditions (long night shifts and working hours during COVID-19 pandemic) fell behind those high rates. Since work fatigue was shown to affect work and wellbeing of doctors as well as their medical performance [65], reducing work fatigue seems mandatory for the wellbeing of Lebanese doctors.

## Data Availability

The datasets generated and/or analysed during the current study are not publicly available due restrictions imposed by the ethics committee but are available from the corresponding author on reasonable request.

## Abbreviations

3D-WFI: 3D-Work Fatigue Inventory
RMSEA: Root Mean Square Error of Approximation
TLI: Tucker Lewis Index
CFI: Comparative Fit Index
A-COPSOQ: Copenhagen Psychosocial Questionnaire
PHQ: Patient Health Questionnaire
